# Security Analysis of Sending or Not-Sending Twin-Field Quantum Key Distribution with Weak Randomness

**DOI:** 10.3390/e24101339

**Published:** 2022-09-23

**Authors:** Xiao-Lei Jiang, Yang Wang, Yi-Fei Lu, Jia-Ji Li, Chun Zhou, Wan-Su Bao

**Affiliations:** 1 Henan Key Laboratory of Quantum Information and Cryptography, SSF IEU, Zhengzhou 450001, China; 2 Synergetic Innovation Center of Quantum Information and Quantum Physics, University of Science and Technology of China, Hefei 230026, China; 3 National Laboratory of Solid State Microstructures, School of Physics and Collaborative Innovation Center of Advanced Microstructures, Nanjing University, Nanjing 210093, China

**Keywords:** twin-field quantum key distribution, weak randomness, asymptotic cases, finite-key

## Abstract

Sending-or-not sending twin-field quantum key distribution (SNS TF-QKD) has the advantage of tolerating large amounts of misalignment errors, and its key rate can exceed the linear bound of repeaterless quantum key distribution. However, the weak randomness in a practical QKD system may lower the secret key rate and limit its achievable communication distance, thus compromising its performance. In this paper, we analyze the effects of the weak randomness on the SNS TF-QKD. The numerical simulation shows that SNS TF-QKD can still have an excellent performance under the weak random condition: the secret key rate can exceed the PLOB boundary and achieve long transmission distances. Furthermore, our simulation results also show that SNS TF-QKD is more robust to the weak randomness loopholes than the BB84 protocol and the measurement-device-independent QKD (MDI-QKD). Our results emphasize that keeping the randomness of the states is significant to the protection of state preparation devices.

## 1. Introduction

Quantum key distribution (QKD) has been widely proved to have information-theoretical security, which is guaranteed by the laws of physics between two authorized users, Alice and Bob [1,2]. However, it is well known that the imperfections of practical devices will compensate the security of the generated key. In fact, some quantum attacks have been discovered and demonstrated by exploiting these imperfections of practical devices. An eavesdropper (Eve) could take advantage of any imperfections in practical system to collect secret information without being discovered, with methods such as wavelength attack [3], the detector control attack [4,5], and the Trojan horse attack [6,7]. Therefore, researchers have to propose corresponding countermeasures to deal with these security threats.

In order to remove side-channel attacks at detection, Lo et al. proposed [8] the measurement-device-independent QKD (MDI-QKD) protocol, while the key rate of the MDI-QKD cannot be better than the linear scale of the channel transmittance. Fortunately, the twin-field QKD (TF-QKD) [9] and the asynchronous MDI-QKD [10] were proposed and improved the key rate to the square root of the channel transmittance. The key rate of them performs $R∼O\sqrt{\eta }$ (where η is the channel transmittance) and it can exceed the Pirandola–Laurenza–Ottaviani–Banchi (PLOB) bound [11]. But the later announcement of the phase information in the original TF-QKD [9] may cause security loopholes [12], so many variants of TF-QKD have been proposed [12,13,14,15] to deal with these loopholes. Particularly, the sending-or-not-sending (SNS) TF-QKD [12], as an efficient protocol, can tolerate large misalignment errors even up to 35% in the single-photon interference. In fact, the SNS TF-QKD protocol has made significant progress in theory [16,17,18,19,20,21,22,23,24,25]. In addition, several experiments on the SNS protocol have also been performed so far [26,27,28,29,30,31].

In a practical QKD system, Eve may shift their target to quantum state preparation devices so that the bit encoding and measurement basis selection are non-randomly modulated by Alice or Bob [32]. For the quantum state preparation vulnerability of the weak randomness, Li et al. proposed [33] a weak randomness model in the BB84 protocol. Under the model, the quantum states that Alice has prepared are divided into two parts: the random part and the non-random part, and the latter may lead to the leakage of information. Since then, the model has been further promoted and applied in other protocols [34,35,36].

In this paper, we generalize the weak randomness attack model to the SNS TF-QKD. In fact, the SNS TF-QKD possesses the property of measurement-device-independent and can be applied using the coherent source with the decoy-state method [37,38,39]. Moreover, the operation of sending or not sending states for Alice and Bob can be regarded as the bit encoding operation, and the operation of selecting time windows can be regarded as the basis selection operation [12]. We analyze the effect of weak randomness on the final security key in the asymptotic and the finite-key size cases. Firstly, we will analytically derive the secret key rate formula based on the weak randomness model in the asymptotic case, and we then calculate the lower bound of the counting rate of the single-photon states and the upper bound of the phase error rate in finite-key cases [16]. In the security analysis of SNS TF-QKD with the weak randomness, we assume that Eve can interfere the quantum state preparation operation, and Eve is responsible for all weak randomness mentioned above. We also assume that the hidden variables ξ and ζ from Eve may determine the quantum states prepared by Alice and Bob, where ξ determines Alice’s quantum states and ζ determines Bob’s quantum states. The probability of non-random quantum states prepared by Alice is $p_{1}$ and the probability of random quantum states is $1-p_{1}$. The probability of non-random quantum states prepared by Bob is $p_{2}$ and the probability of random quantum states is $1-p_{2}$. If $p_{1}=1$ or $p_{2}=1$, apparently, Eve can acquire all the information, that is, R=0. If $p_{1}=p_{2}=0$, Eve may partly acquire information. If $0<p_{1}<1,0<p_{2}<1$, the weak randomness model could be applied to quantify the maximal amount of leaked information and explore the security of SNS TF-QKD with weak randomness. Using the experimental parameters, we demonstrate that the secret key rate of SNS TF-QKD still can exceed the PLOB bound and achieve long secure transmission distances under the weak random condition. We then compare the effect of weak randomness on the BB84 protocol and MDI-QKD protocol, and we deduce that SNS TF-QKD can tolerate more weak randomness vulnerability.

The rest of paper is organized as follows: we describe a four-intensity decoy-state SNS TF-QKD protocol in Section 2. In Section 3, we analyze the effects of the weak randomness on the SNS TF-QKD protocol in the asymptotic and of the finite-key size cases. The numerical simulations are shown in Section 4 and the conclusion is made in Section 5.

## 2. Protocol Description

In the practical QKD system, we usually choose the weak coherent state source instead of the single photon source. Here, we consider the four-intensity decoy-state SNS protocol [16], and the description of the protocol is presented as follows:

1. Preparation. At any times window *i*, Alice (Bob) independently determines whether it is a decoy window or a signal window with probabilities $p_{x}$ and $p_{z}$. If it is a decoy window, Alice (Bob) sends out to Charlie a decoy pulse in a phase-randomized coherent state $|\sqrt{\mu_{a}}e^{i\delta_{A}}\rangle$, $|\sqrt{\mu_{b}}e^{i{\delta^{\prime }}_{A}}\rangle$ or $|0\rangle$ ($|\sqrt{\mu_{a}}e^{i\delta_{B}}\rangle$, $|\sqrt{\mu_{b}}e^{i{\delta^{\prime }}_{B}}\rangle$ or $|0\rangle$) with probabilities of $p_{\mu_{a}}$, $p_{\mu_{b}}$, $p_{0}$. We suppose $\mu_{a}<\mu_{b}$. If it is a signal window, Alice (Bob) decides to send out to Charlie a signal pulse in phase-randomized coherent states $|\sqrt{\mu_{z}}e^{i\delta_{A}}\rangle$ or a vacuum state $|0\rangle$ ($|\sqrt{\mu_{z}}e^{i\delta_{B}}\rangle$ or $|0\rangle$) with probabilities of $p_{z0}$ and $1-p_{z0}$, where $\delta_{A(B)}$ is random in $\left[0,2\pi \right)$. Here, we assume that consecutive photons are well-separated in the decoy and the signal time windows. Note that a coherent state of intensity μ and global phase δ is a linear superposition of photon-number states $|\sqrt{\mu }e^{i\delta }\rangle =\sum_{k=0}^{\infty }\frac{e^{-\mu /2}{\left(\sqrt{\mu }e^{i\delta }\right)}^{k}}{\sqrt{k!}}|k\rangle$. Whenever Alice or Bob sends a coherent state of intensity μ, it can be equivalently regarded as a probabilistic mixture of different photon-number states $\int_{0}^{2\pi }|\sqrt{\mu }e^{i\delta }\rangle \langle \sqrt{\mu }e^{i\delta }|d\delta /2\pi =\sum_{k=0}^{\infty }\frac{e^{-\mu }\mu^{k}}{k!}|k\rangle \langle k|$.
2. Measurement. Alice and Bob send the chosen states to Charlie. Charlie then performs interferometric measurements on the incoming quantum signals after taking phase compensation and announces the measurement results of which detector clicks to Alice and Bob. An effective event is defined as follows: (1) if only one detector clicks corresponding to a time window *i* when both Alice and Bob have determined the signal window, it is defined as an effective event. (2) If only one detector clicks corresponding to a time window *i* when both Alice and Bob have determined a decoy window and sent the coherent states with the same intensity, and in that time window, the pre-chosen values $\delta_{A}$ and $\delta_{B}$ satisfy post-selection criterion, which is:
  (1) $$1-\left|\cos(\delta_{A}-\delta_{B}-\psi_{AB})\right|\leq \left|\lambda \right|,$$
  where $\delta_{A}$ and $\delta_{B}$ are the random phases of coherent states prepared by Alice and Bob, respectively. $\psi_{AB}$ could take an arbitrary value and it is set properly to acquire a satisfactory key rate, which will be different from time to time due to phase drift. The value of λ is determined by the size of the phase slice Δ, which is chosen by Alice and Bob. In fact, Equation (1) is equivalent to:
  (2) $$\left|\theta_{A}-\theta_{B}-\psi_{AB}\right|\leq \frac{\Delta }{2},\left|\theta_{A}-\theta_{B}-\psi_{AB}-\pi \right|\leq \frac{\Delta }{2}.$$
  where $\left|x\right|$ represents the minor angle enclosed by two rays, which enclose the rotational angle.
3. Sifting. Alice and Bob announce decoy windows and signal windows of each other. If both Alice and Bob choose the decoy window, it is defined to be an $\tilde{X}$ window. If both Alice and Bob choose the signal window, it is defined to be a *Z* window. In an $\tilde{X}$ window, it is an $X_{1}$ window, which is a subset of $\tilde{X}$ windows, when they choose the same intensity $\mu_{a(b)}$. Additionally, it is an $X_{0}$ window when Alice (Bob) determines a signal window, while Bob (Alice) determines a decoy window, or when both Alice and Bob determine the decoy window, but choose different intensities. According to the effective events criterion introduced above, Alice and Bob decide whether one-detector clicks event is an effective event. We define three kinds of sets: Z, $X_{1}$ and $X_{0}$, which include all effective events in *Z*, $X_{1}$ and $X_{0}$ windows.
4. Parameter estimation. For the events in the set Z of the *Z* window, if Alice decides to send out a phased-randomized weak coherent state, she (he) denotes a bit 1 and if she (he) decides to send a vacuum state, she (he) denotes a bit 0. If Bob decides to send out a phased-randomized weak coherent state, she (he) denotes a bit 0 and if she (he) decides not to send a vacuum state, she (he) denotes a bit 0. We notice that it is the decision that determines the bit value rather than what they send. Then, Alice and Bob could obtain the $n_{t}$ bit strings, and they will get an error bit if an effective event happens when both Alice and Bob decide to send or not send. Finally, adopting the decoy-state method, Alice and Bob could estimate the number of the single-photon states $n_{1}$ and phase-flip error rate $e_{1}^{ph}$ according to the events in Z windows. They could estimate the lower bound of $n_{1}$ and upper bound of $e_{1}^{ph}$ according to the events in $X_{1}$ and $X_{0}$ windows.
5. Error correction. Alice and Bob perform an error correction scheme to correct bit strings obtained in the last step. To achieve this goal, it consumes at most $leak_{EC}$ bits of error correction data. Then, Alice and Bob exploit a random two-universal hash function to carry out an error verification operation, which Alice sends a hash of length $\log_{2}\left(1/\varepsilon_{cor}\right)$ to make sure that the key bits of Alice are the same as Bob.
6. Private amplification. In order to reduce Eve’s information of final keys, Alice and Bob exploit the random two-universal hash function to extract two shorter strings of length *l*. Finally, Alice and Bob obtain the secret key strings $S_{A}$ and $S_{B}$.

## 3. Security Analysis

In this section, we may analyze the effects of the weak randomness on the decoy-state SNS TF-QKD in the asymptotic and the finite-key size cases. We may derive concise formulas for estimating the lower bound of the single-photon yield and the upper bound of the phase-flip error rate.

### 3.1. Parameter Estimation in the Asymptotic Case

As discussed above, the effective events that Alice decides to send and Bob decides not to send, or Alice decides not to send and Bob decides to send, in *Z* windows could generate the secret key. As a matter of fact, the selection of signal windows and decoy windows can be considered as the basis selection. In the decoy windows or the signal windows, sending or not sending a phase-randomized coherent state can be considered as the bit encoding. This assumption is reasonable by considering two cases. The first one is that the random numbers may be leaked to Eve because of the imperfection of the random number generator devices. The other one is that the imperfect state modulation may be prepared by different laser diodes from Alice and Bob, and they can be partly distinguished through observing the properties of the spectrum and timing sequence. Therefore, the weak randomness attack model which is used in the BB84 protocol and the MDI-QKD protocol is still appropriate to the SNS TF-QKD protocol. Under the weak randomness model, we suppose that the quantum states prepared by Alice and Bob can be considered as the set *S* and *T*, and $\left|S\right|$ and $\left|T\right|$ represent the number of elements of the set *S* and *T*. For the set of quantum states prepared by Alice (Bob), $S_{1}$($T_{1}$) is the random part and $S_{2}$($T_{2}$) is the non-random part. At this time, the probability of a non-random parameter at Alice could be defined as $p_{1}=\frac{\left|S_{2}\right|}{\left|S\right|}$, and the probability of non-random parameter at Bob could be defined as $p_{2}=\frac{\left|T_{2}\right|}{\left|T\right|}$. Under the practical QKD system, although we can assume the quantum devices at Alice and Bob are identical, the attack capabilities of Eve against Alice and Bob cannot be guaranteed same. That is, $p_{1}=p_{2}$ is not necessary. In the model, we can re-describe the quantum states prepared by Alice and Bob in the practical system:

(3) $$\rho_{\mathrm{Alice}}^{\prime }=\frac{p_{1}}{2}\sum_{a=0,1}|a\rangle {\langle a|}_{Alice}⊗|a\rangle {\langle a|}_{Eve}+\left(1-p_{1}\right)\rho_{Alice}⊗|2\rangle {\langle 2|}_{Eve},$$

(4) $$\rho_{\mathrm{Bob}}^{\prime }=\frac{p_{2}}{2}\sum_{b=0,1}|b\rangle {\langle b|}_{Bob}⊗|b\rangle {\langle b|}_{Eve}+\left(1-p_{2}\right)\rho_{Bob}⊗|2\rangle {\langle 2|}_{Eve}.$$

where the quantum states prepared by Alice and Bob can be divided into two parts: the first part is prepared by a non-random set, and the second part is prepared by a random set. In the case in which the quantum states are in the first part, the assistant quantum states of Eve are related to Alice’s (Bob’s) system. More precisely, if the auxiliary quantum state of Eve is $|a\rangle {\langle a|}_{Eve}$, Eve can obtain the secret key *a* of Alice; if the auxiliary quantum state of Eve is $|b\rangle {\langle b|}_{Eve}$, Eve can obtain the secret key *b* of Bob. In the case in which the quantum state is prepared in the second part, if the auxiliary quantum state of Eve is $|2\rangle {\langle 2|}_{Eve}$, it indicates that Alice and Bob prepared the phase-randomized coherent states, which is equivalent to a probabilistic mixture of different photon-number states $\rho_{Alice}=\sum_{k=0}^{\infty }\frac{e^{-\mu_{a}}{\mu_{a}}^{k}}{k!}|k\rangle \langle k|$ and $\rho_{Bob}=\sum_{k=0}^{\infty }\frac{e^{-\mu_{b}}{\mu_{b}}^{k}}{k!}|k\rangle \langle k|$ and Eve can not distinguish encoding states. Therefore, Eve can distinguish the random part and non-random part states of Alice and Bob by observing auxiliary quantum states. The practical QKD systems require perfect random numbers for preparing quantum states. Unfortunately, the weak randomness of state preparation in practical QKD systems is universal because of the imperfections of quantum devices.

Under the weak randomness model, Eve wants to get more information, so she (he) may perform the attenuation operation on the quantum states from a random part by a certain probability, but cannot perform that on the quantum states from a non-random part. The attacker’s attenuation operation increases the non-randomness of the quantum states reaching Charlie, which leads to the increasing of the amount of information controlled by Eve. Because of the attenuation operation, we can assume that the bit error rate only happens in the random part, while the non-random part does not produce bit errors. As long as Eve controls the attenuation to make the final error rate less than a reasonable value, Alice and Bob cannot detect the presence of Eve, so Eve implements a weak-random attack. In this case, the non-random probability on Charlie’s side can be amplified by considering the signal loss so that the maximal transmission distance may be seriously decreased and the single photon counting rate in *Z* windows $s_{1}^{Z}$, which is used to generate the secret key, decreases, and the bit error rate in *X* windows $e_{1}^{X}$ increases. Then, we estimate the parameters within the effects of weak randomness on SNS TF-QKD in the asymptotic case.

Firstly, we may analyze the state preparation step under the weak randomness condition. In the case where both Alice and Bob choose a signal window, Alice then sends quantum states and Bob does not send quantum states, or Bob sends quantum states and Alice does not send quantum states, and the probability of the states prepared by Alice or Bob with randomness is $2p_{z0}\left(1-p_{z0}\right)\mu_{z}e^{-\mu_{z}}\left(1-p_{1}\right)\left(1-p_{2}\right)$, and the probability with non-randomness is $2p_{z0}\left(1-p_{z0}\right)\mu_{z}e^{-\mu_{z}}\left(1-\left(1-p_{1}\right)\left(1-p_{2}\right)\right)=2p_{z0}\left(1-p_{z0}\right)\mu_{z}e^{-\mu_{z}}\left(p_{1}+p_{2}-p_{1}p_{2}\right)$. Here, the probability of quantum states with non-randomness prepared by Alice is $p_{1}$, and the probability of quantum states with non-randomness prepared by Bob is $p_{2}$. In the practical QKD system, Eve can control the attenuation of the quantum states in the channel, and only attenuates the quantum states of the random part to ensure that the non-random part of the quantum states reach Charlie without attenuation. At the Charlie’s side, the proportion of non-random quantum states from the non-random part increases, and the proportion of quantum states from the random part decreases. In order to acquire more information, Eve may make the non-random scale of Charlie as large as possible. To ensure that she (he) may not be detected by both communicators, Eve must control the probability of attenuation or the attack will fail. Here, we calculate the probability of signal loss of the coherent states from the random set:

(5) $$\;p_{loss1}=2p_{z0}\left(1-p_{z0}\right)\mu_{z}e^{-\mu_{z}}\frac{s_{1}^{Z}-\left(p_{1}+p_{2}-p_{1}p_{2}\right)}{1-(p_{1}+p_{2}-p_{1}p_{2})},$$

From the Equation (5), we can conclude the proportion of quantum states reaching Charlie with non-randomness:

(6) $$p_{non-rand1}=\frac{p_{1}+p_{2}-p_{1}p_{2}}{s_{1}^{Z}},$$

The proportion of quantum states reaching Charlie with randomness is:

(7) $$p_{rand1}=\frac{s_{1}^{Z}-\left(p_{1}+p_{2}-p_{1}p_{2}\right)}{s_{1}^{Z}}.$$

Since the quantum states of the random part are attenuated, the effective counting rate $s_{1}^{Z}$ in the *Z* window and the bit error rate $e_{1}^{X}$ in the X window will change. In fact, the quantum states of the non-random set cannot generate the security key, and only the quantum states of the random set may generate the security key. The counting rate from the non-random set in *Z* windows that cannot generate the secret key is:

(8) $${\tilde{s}}_{1}^{Z}=2p_{z0}\left(1-p_{z0}\right)\mu_{z}e^{-\mu_{z}}\left(p_{1}+p_{2}-p_{1}p_{2}\right),$$

The counting rate from the random set in *Z* windows that can generate the secret key satisfies:

(9) $$s_{1}^{\prime }=s_{1}^{Z}-{\tilde{s}}_{1}^{Z}.$$

Under the weak randomness condition, in order to obtain more information, Eve attenuates the random part of the quantum states to ensure that the final error code only comes from the quantum states of the random part. The bit error rate in $\tilde{X}$ windows after the attenuation operation is calculated:

(10) $$e_{1}^{\prime }=\frac{e_{1}^{X}}{p_{rand1}}=\frac{e_{1}^{X}s_{1}^{Z}}{s_{1}^{Z}-\left(p_{1}+p_{2}-p_{1}p_{2}\right)},$$

Alice and Bob use the announced data from $X_{1}$ windows to calculate the counting rate $s_{1}^{Z}$, which is also the value for *Z* windows. The number of bits generated in *Z* windows could be calculated from this value. Moreover, the error rate of bits in $X_{1}$ windows of intensity μ and $E_{\mu }^{X}$, the counting rate of intensity μ and $S_{\mu }$, and the counting rate of vacuum $s_{0}$ can be observed, so we can calculate the upper bound value of the flipping rate [12]:

(11) $$e_{1}^{X}\leq e_{1}^{X,U}=\frac{S_{\mu }E_{\mu }^{X}-e^{-2\mu }s_{0}/2}{2\mu e^{-2\mu }s_{1}^{Z}}.$$

In the asymptotical condition, the phase-flip rate satisfies $e_{1}^{ph}=e_{1}^{X}$. Similarly, under the weak randomness model, the phase-flip rate satisfies $e_{1}^{ph}=e_{1}^{\prime }$. Finally, we can distill the final key with an asymptotic key rate formula with weak randomness:

(12) $$R=2p_{z0}\left(1-p_{z0}\right)\mu_{z}e^{-\mu_{z}}s_{1}^{\prime }\left[1-H(e_{1}^{ph})\right]-fS_{Z}H\left(E_{Z}\right).$$

where $H\left(x\right)=-x\log_{2}\left(x\right)-\left(1-x\right)\log_{2}\left(1-x\right)$ is the binary entropy function, $S_{Z}$ is the observed counting rate of *Z* windows, $E_{Z}$ is the corresponding bit-flip error rate and *f* is the efficiency of error correction.

### 3.2. Parameter Estimation with the Finite-Key Size

In a practical QKD system, the number of photons sent is finite and the intensities cannot be infinite in *Z* windows or $\tilde{X}$ windows. In this section, we consider the effects of the weak randomness on SNS TF-QKD with the finite-key size based on the universally composable framework [40]. To close the gap between the expected values and observed values, we exploit the Improved-Chernoff bound [41,42,43] to estimate the counting rate of single-photon states $n_{1}$ and the phase-flip error rate $e_{1}^{ph}$.

Firstly, we make an introduction of the universally composable framework [40]:

**Definition** **1.** *If the final key strings $S_{A}$ and $S_{B}$ of Alice and Bob satisfy the following conditions, the protocol is defined to be ε-secure:*

- *Correctness. A protocol is $\varepsilon_{cor}$-correct if $S_{A}$ and $S_{B}$ of Alice and Bob are not identical with the maximal probability of $\varepsilon_{cor}$:*
$$\mathrm{Pr}\left(S_{A}\neq S_{B}\right)\leq \varepsilon_{cor},$$
- *Secrecy. The final key strings S ($S_{A}$ or $S_{B}$) are said to be $\varepsilon_{\sec}$-secret with respect to the Eve holding a quantum system E if:*
$$\frac{1}{2}p_{abort}∥\rho_{S}-\rho_{U}⊗\rho_{E}∥\leq \varepsilon_{\sec},$$
  *where $p_{abort}$ denotes the probability of protocol failure aborted, $\rho_{S}$ denotes the classical-quantum states of the system for Alice (Bob) and system E, and $\rho_{U}$ denotes the fully mixed states on $S_{A}$ or $S_{B}$.*

The security against general attacks based on the entropic uncertainty relation for the smooth min-extropy in the SNS TF-QKD has been proven. According to the finite-key analysis based on the universally composable framework, the length of secret keys can be presented as follows [16,44]:

(13) $$\ell =n_{1}\left[1-H(e_{1}^{ph})\right]-leak_{EC}-\log_{2}\frac{2}{\varepsilon_{cor}}-2\log_{2}\frac{1}{\sqrt{2}\varepsilon_{PA}\hat{\varepsilon }}.$$

According to the composable framework, the security coefficient of the whole protocol is $\varepsilon_{tol}=\varepsilon_{cor}+\varepsilon_{\sec}$, where $\varepsilon_{\sec}=2\hat{\varepsilon }+4\bar{\varepsilon }+\varepsilon_{PA}+\varepsilon_{n1}$. $\varepsilon_{cor}$ is the failure probability of error correction; $\bar{\varepsilon }$ and $\varepsilon_{PA}$ are the failure probability for the estimation of the phase-flip error rate and privacy amplification; $\varepsilon_{n1}$ is the failure probability for estimation of the lower bound of the counting rate of single-photon states. $leak_{Ec}=fn_{t}h\left(E_{z}\right)$, where $n_{t}$ is final length of the secret key string, $E_{z}$ is the corresponding error rate.

In $\tilde{X}$ windows, Alice and Bob do not announce any phase information. The coherent states sent out can be regarded as a classical mixture of different photon numbers. We denote $\rho_{v}$, $\rho_{a}$ and $\rho_{b}$. Let $N_{\alpha \beta }$ be the number of the events which Alice sends $\rho_{\alpha }$ and Bob sends $\rho_{\beta }$, where $\left(\alpha ,\beta \right)\in \left\{\left(v,v\right),\left(v,a\right),\left(a,v\right),\left(v,b\right),\left(b,v\right)\right\}$. Here, we suppose that Alice and Bob repeat the Preparation and Measurement step *N* times, so $N_{\alpha \beta }$ can be expressed as follows [16]:

(14) $$N_{vv}=\left[{\left(1-p_{\mu_{a}}-p_{\mu_{a}}\right)}^{2}{\left(1-p_{z}\right)}^{2}+2\left(1-p_{\mu_{a}}-p_{\mu_{b}}\right)\left(1-p_{z}\right)p_{z}p_{z0}\right]N,$$

(15) $$N_{va}=N_{av}=\left[\left(1-p_{\mu_{a}}-p_{\mu_{b}}\right){\left(1-p_{z}\right)}^{2}p_{\mu_{a}}+\left(1-p_{z}\right)p_{z}p_{z0}p_{\mu_{a}}\right]N,$$

(16) $$N_{vb}=N_{bv}=\left[{\left(1-p_{z}\right)}^{2}\left(1-p_{\mu_{a}}-p_{\mu_{b}}\right)p_{\mu_{b}}+\left(1-p_{z}\right)p_{z}p_{z0}p_{\mu_{b}}\right]N.$$

let $n_{\alpha \beta }$ be the number of effective events of one-detector heralded corresponding to the $N_{\alpha \beta }$. For $\left(\alpha ,\beta \right)\in \left\{\left(v,v\right),\left(v,a\right),\left(a,v\right),\left(v,b\right),\left(b,v\right)\right\}$, $n_{\alpha \beta }$ can be expressed as:

(17) $$n_{vv}=2p_{d}\left(1-p_{d}\right)N_{vv},$$

(18) $$n_{va}=n_{av}=2\left[\left(1-p_{d}\right)e^{-\eta \mu_{a}/2}-{\left(1-p_{d}\right)}^{2}e^{-\eta \mu_{a}}\right]N_{va},$$

(19) $$n_{vb}=n_{bv}=2\left[\left(1-p_{d}\right)e^{-\eta \mu_{b}/2}-{\left(1-p_{d}\right)}^{2}e^{-\eta \mu_{b}}\right]N_{vb}.$$

To close the gap between the expected values and observed values, we apply Improved-Chernoff bound [41,42,43] to obtain the upper and lower bound of the expected value of $n_{\alpha \beta }$ considering independent event conditions:

(20) $$\langle n_{\alpha \beta }^{U}\rangle =\frac{n_{\alpha \beta }}{1-\delta_{U}},\langle n_{\alpha \beta }^{L}\rangle =\frac{n_{\alpha \beta }}{1+\delta_{L}}.$$

where we can obtain the values of $\delta_{U}$ and $\delta_{L}$ by solving the following equations:

(21) $${\left[\frac{e^{-\delta_{U}}}{{\left(1-\delta_{U}\right)}^{1-\delta_{U}}}\right]}^{X/(1-\delta_{U})}=\frac{\varepsilon }{2},$$

(22) $${\left[\frac{e^{\delta_{L}}}{{\left(1+\delta_{L}\right)}^{1+\delta_{L}}}\right]}^{X/(1+\delta_{L})}=\frac{\varepsilon }{2}.$$

where ε is the failure probability, $\langle x\rangle$ is the expected value of *x*. From Equations (20)–(22), we can obtain the upper and lower bound of $n_{\alpha \beta }$, $\langle n_{\alpha \beta }^{U}\rangle$ and $\langle n_{\alpha \beta }^{L}\rangle$. Then, we denote the counting rate of state $\rho_{\alpha }$ and state $\rho_{\beta }$ as $S_{\alpha \beta }$, which can be expressed as:

(23) $$S_{\alpha \beta }=\frac{n_{\alpha \beta }}{N_{\alpha \beta }},$$

Obviously, from Equations (20) and (23), we can easily obtain the upper and lower bound of the expected value of $S_{\alpha \beta }$:

(24) $$\langle S_{\alpha \beta }^{U}\rangle =\frac{\langle n_{\alpha \beta }^{U}\rangle }{N_{\alpha \beta }},\langle S_{\alpha \beta }^{L}\rangle =\frac{\langle n_{\alpha \beta }^{L}\rangle }{N_{\alpha \beta }}.$$

Similarly, in the case of the finite-key size, the probability of signal loss of the coherent states from the random set can be calculated as:

(25) $$p_{loss2}=\frac{n_{1}-2p_{z0}\left(1-p_{z0}\right)\mu_{z}e^{-\mu_{z}}\left(p_{1}+p_{2}-p_{1}p_{2}\right)N}{N-(p_{1}+p_{2}-p_{1}p_{2})N},$$

from the Equation (25), we can conclude the proportion of quantum states reaching Charlie with non-randomness:

(26) $$p_{non-rand2}=\frac{2p_{z0}\left(1-p_{z0}\right)\mu_{z}e^{-\mu_{z}}\left(p_{1}+p_{2}-p_{1}p_{2}\right)N}{n_{1}},$$

the proportion of quantum states reaching Charlie with randomness is:

(27) $$p_{rand2}=\frac{n_{1}-2p_{z0}\left(1-p_{z0}\right)\mu_{z}e^{-\mu_{z}}\left(p_{1}+p_{2}-p_{1}p_{2}\right)N}{n_{1}}.$$

Due to the quantum states in the random part being attenuated, the number of the effective counting rate $n_{1}$ and the phase-flip error rate $e_{1}^{ph}$ in the *Z* window may change. The quantum states in the non-random set cannot generate the security key, and only the quantum states in the random set may generate the security key. The number of the effective events caused by single-photon states from the non-random set in *Z* windows that cannot generate the secret key is:

(28) $${\tilde{n}}_{1}=2p_{z0}\left(1-p_{z0}\right)\mu_{z}e^{-\mu_{z}}{\tilde{s}}_{1}^{Z}N,$$

The number of the effective events caused by single-photon states from the random set that can generate the secret key is:

(29) $$n_{1}^{\prime }=n_{1}-{\tilde{n}}_{1}=2p_{z0}\left(1-p_{z0}\right)\mu_{z}e^{-\mu_{z}}\left(s_{1}^{Z}-{\tilde{s}}_{1}^{Z}\right)N.$$

where the lower bound of the effective counting rate $s_{1}^{Z}$ of the finite-key size satisfies [16,17]:

(30) $$\langle s_{1}^{Z}\rangle \geq \langle s_{1}^{Z,L}\rangle =\frac{1}{2\mu_{a}\mu_{b}\left(\mu_{b}-\mu_{a}\right)}\left[{\mu_{b}}^{2}e^{\mu_{a}}\left(\langle S_{va}^{L}\rangle +\langle S_{av}^{L}\rangle \right)-{\mu_{a}}^{2}e^{\mu_{b}}\left(\langle S_{vb}^{U}\rangle +\langle S_{bv}^{U}\rangle \right)\right.\left.-2\left({\mu_{b}}^{2}-{\mu_{a}}^{2}\right)\langle S_{vv}^{U}\rangle \right].$$

Moreover, in order to estimate the upper bound of $e_{1}^{ph}$, we need to define two new subsets $C_{\Delta }^{+}$ and $C_{\Delta }^{-}$ of $X_{1}$ windows when $\left|\delta_{A}-\delta_{B}\right|\leq \frac{\Delta }{2}$ and $\left|\delta_{A}-\delta_{B}-\pi \right|\leq \frac{\Delta }{2}$, where we have supposed that $\psi_{AB}=0$. The number of instances in $C_{\Delta }^{+}$ and $C_{\Delta }^{-}$ is:

(31) $$N_{\Delta^{+}}=N_{\Delta^{-}}=\frac{\Delta }{2\pi }{\left(1-p_{z}\right)}^{2}{p_{\mu_{a}}}^{2}N,$$

Here, we denote the number of effective events of right detector from $C_{\Delta }^{+}$ and the number of effective events of left detector from $C_{\Delta }^{-}$ as $n_{\Delta^{+}}^{R}$ and $n_{\Delta^{-}}^{L}$:

(32) $$n_{\Delta^{+}}^{R}=\left(W_{a}\left(1-e_{d}\right)+C_{a}e_{d}\right)N_{\Delta }^{+},$$

(33) $$n_{\Delta^{-}}^{L}=\left(W_{a}\left(1-e_{d}\right)+C_{a}e_{d}\right)N_{\Delta }^{-}.$$

where $W_{a}$ and $C_{a}$ is the average probability of correct counting and wrong counting, respectively, which can be given as:

(34) $$W_{a}=\frac{1}{\Delta }\int_{-\Delta /2}^{\Delta /2}\left(1-p_{d}\right)e^{-2\eta \mu_{a}\sin^{2}\frac{\theta }{2}}d\theta -{\left(1-p_{d}\right)}^{2}e^{-2\eta \mu_{a}},$$

(35) $$C_{a}=\frac{1}{\Delta }\int_{-\Delta /2}^{\Delta /2}\left(1-p_{d}\right)e^{-2\eta \mu_{a}\cos^{2}\frac{\theta }{2}}d\theta -{\left(1-p_{d}\right)}^{2}e^{-2\eta \mu_{a}}.$$

Considering independent event conditions, we apply the Improved-Chernoff bound [41,42,43] to obtain the upper and lower bound of the expected value of $n_{\Delta^{\pm }}^{R}$. For the finite sample sizes, the number of effective events of the right and left detector from $C_{\Delta }^{+}$ and $C_{\Delta }^{-}$ satisfies:

(36) $$\langle n_{\Delta^{+}}^{R,U}\rangle =\frac{n_{\Delta^{+}}^{R}}{1-{\delta^{\prime }}_{U}},\langle n_{\Delta^{+}}^{R,L}\rangle =\frac{n_{\Delta^{+}}^{R}}{1+{\delta^{\prime }}_{L}},$$

(37) $$\langle n_{\Delta^{-}}^{L,U}\rangle =\frac{n_{\Delta^{-}}^{L}}{1-{\delta^{\prime }}_{U}},\langle n_{\Delta^{-}}^{L,L}\rangle =\frac{n_{\Delta^{-}}^{L}}{1+{\delta^{\prime }}_{L}}.$$

With the failure probability ε, we can obtain the values of ${\delta^{\prime }}_{U}$ and ${\delta^{\prime }}_{L}$ by solving the following equations:

(38) $${\left[\frac{e^{-{\delta^{\prime }}_{U}}}{{\left(1-{\delta^{\prime }}_{U}\right)}^{1-{\delta^{\prime }}_{U}}}\right]}^{X/(1-{\delta^{\prime }}_{U})}=\frac{\varepsilon }{2},$$

(39) $${\left[\frac{e^{{\delta^{\prime }}_{L}}}{{\left(1+{\delta^{\prime }}_{L}\right)}^{1+{\delta^{\prime }}_{L}}}\right]}^{X/(1+{\delta^{\prime }}_{L})}=\frac{\varepsilon }{2}.$$

We have the upper bound of the expected value of counting error rate from $C_{\Delta }^{+}$ and $C_{\Delta }^{-}$:

(40) $$\langle T_{\Delta }^{U}\rangle =\frac{1}{2}\left(\langle T_{\Delta^{+}}^{U}\rangle +\langle T_{\Delta^{-}}^{U}\rangle \right)=\frac{1}{2}\left(\frac{\langle n_{\Delta^{+}}^{R,U}\rangle }{N_{\Delta }^{+}}+\frac{\langle n_{\Delta^{-}}^{R,U}\rangle }{N_{\Delta }^{-}}\right),$$

Eve may attenuate the random part of the quantum states to ensure that the final error code only comes from the quantum states in the random part. The value of the counting error rate after the attenuation operation is calculated:

(41) $${T_{\Delta }}^{\prime }=\frac{\langle T_{\Delta }^{U}\rangle }{p_{rand2}}=\frac{\langle T_{\Delta }^{U}\rangle s_{1}^{Z}}{s_{1}^{Z}-\left(p_{1}+p_{2}-p_{1}p_{2}\right)}.$$

The upper bound of the expected value of the phase-flip error rate satisfies [16,17]:

(42) $$\langle e_{1}^{ph}\rangle \leq \langle e_{1}^{ph,U}\rangle =\frac{{T_{\Delta }}^{\prime }-\frac{1}{2}e^{-2\mu_{a}}\langle S_{vv}^{L}\rangle }{2\mu_{a}e^{-2\mu_{a}}\langle s_{1}^{Z,L}\rangle },$$

Furthermore, we are supposed to simulate the information leakage in the protocol. According to the events in *Z* windows, Alice and Bob can obtain a secret string of $n_{s}$ bits. They do not care about which detector clicks as long as only one detector clicks. The length of the secret key string is $n_{t}=n_{signal}+n_{error}$. The number of right bits $n_{signal}$ and wrong bits $n_{error}$ can be given:

(43) $$n_{signal}=4Np_{z}^{2}p_{z0}\left(1-p_{z0}\right)\left[\left(1-p_{d}\right)e^{-\eta \mu_{z}/2}\right],$$

(44) $$n_{error}=2Np_{z}^{2}{\left(1-p_{z0}\right)}^{2}\left[\left(1-p_{d}\right)e^{-\eta \mu_{z}/2}I_{0}\left(\eta \mu_{z}\right)-{\left(1-p_{d}\right)}^{2}e^{-\eta \mu_{z}}\right]+2Np_{z}^{2}p_{z0}^{2}\left(1-p_{d}\right).$$

where $I_{0}\left(x\right)$ is the zero-order hyperbolic Bassel function of the first kind. The error rate of the final key string is $E_{z}=\frac{n_{error}}{n_{t}}$.

Finally, combining the Equations (29), (42)–(44), we can calculate the length of final security key in the SNS TF-QKD protocol with the weak randomness:

(45) $$\ell^{\prime }=n_{1}^{\prime }\left[1-H(e_{1}^{ph})\right]-leak_{EC}-\log_{2}\frac{2}{\varepsilon_{cor}}-2\log_{2}\frac{1}{\sqrt{2}\varepsilon_{PA}\hat{\varepsilon }}.$$

## 4. Numerical Simulations

Here, we simulate the performance of effects of weak randomness on SNS TF-QKD in the asymptotic case and with the finite-key size. We use the linear model to numerically simulate the performance of the protocol. Firstly, we set the experimental parameters that we may exploit. Then, we set the results of the final secret key rate and the analysis of results.

We define $\eta =10^{-\alpha L/10}$ as the fiber transmittance, where α=0.2 (dB/km) is the fiber loss coefficient and *L* is the length of fiber between Charlie and Alice (Bob). $\eta_{d}=80\%$ is the detection efficiency of the relay Charlie, and $p_{d}=10^{-10}$ is the dark count of Charlie’s detectors. The failure probability of statistical fluctuations analysis is fixed to $\varepsilon =10^{-10}$, and f=1.1 is the efficiency of error correction. R=ℓℓNN is the final secret key rate, where *N* is the total number of transmitting signals sent by Alice and Bob. The numerical parameters are listed in Table 1. Here, we set $\varepsilon_{cor}=\hat{\varepsilon }=\varepsilon_{PA}=\varepsilon$, $\bar{\varepsilon }=3\varepsilon$, and $\varepsilon_{n_{1}}=4\varepsilon$.

**Figure 1:**
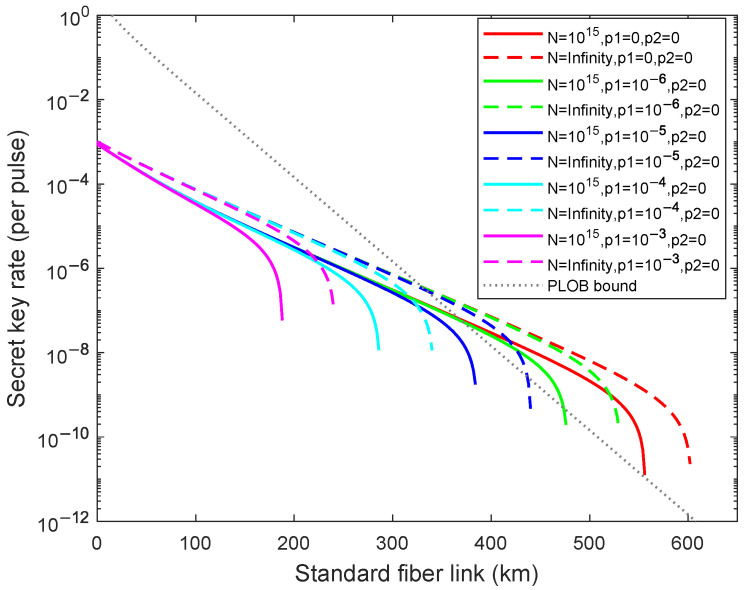
(Color online) The optimal key rate (bits per pulse) in logarithmic scale versus transmission distance between Alice and Bob when the weak randomness exists for only one party (Alice or Bob) $p_{1}=10^{-x}\;\left(x=6,5,4,3\right),p_{2}=0\;$(curves from right to left). The dashed lines are results of the asymptotic case, and the solid lines are the results of the finite-key size $N=10^{15}$. The gray dotted line is the PLOB bound.

**Figure 2:**
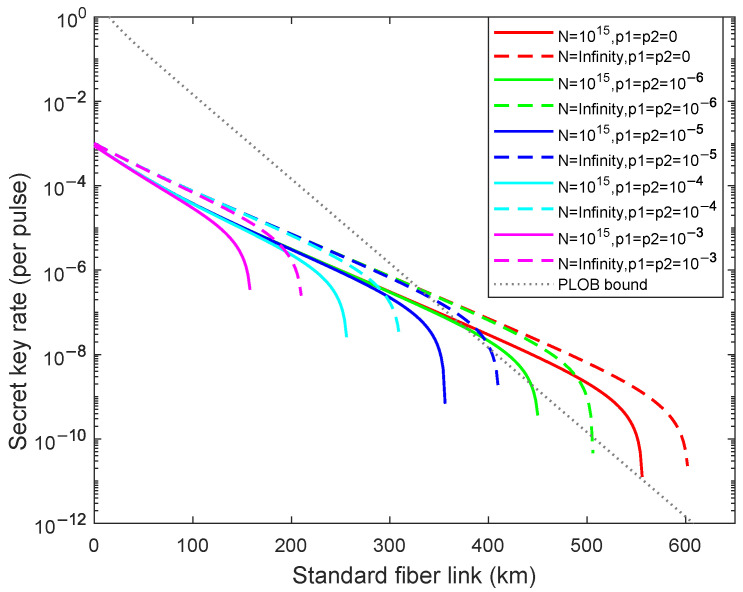
(Color online)The optimal key rate (bits per pulse) in logarithmic scale versus transmission distance between Alice and Bob when the weak randomness exists for two parties $p_{1}=p_{2}=10^{-x}\left(x=6,5,4,3\right)$(curves from right to left). The dashed lines are results of asymptotic cases and the solid lines are the results of the finite-key size $N=10^{15}$. The gray dotted line is the PLOB bound.

Firstly, we analyze the results of weak randomness existing in only one party (Alice or Bob) in the asymptotic and the finite-key size cases in Figure 1. Here, $p_{1}\neq 0,p_{2}=0$ means that Eve just masters the randomness information of Alice, and $p_{1}=0,10^{-x}\left(x=6,5,4,3\right)$ means that Eve has different abilities for controlling the randomness information. We then analyze the results of weak randomness existing in both parties in the asymptotic case and the finite-key size cases in Figure 2, where Eve masters the randomness information of both Alice and Bob. As illustrated in Figure 1 and Figure 2, the dashed lines from right to left are acquired for different weak randomness parameters $p_{1}=0,10^{-6},10^{-5},10^{-4},10^{-3}$ with the infinite number of total pulses, and the solid lines from right to left are acquired for different weak randomness parameters $p_{1}=0,10^{-x}\left(x=6,5,4,3\right)$ with the fixed finite number of total pulses $N=10^{15}$. In the Figure 1, compared with the perfect randomness $p_{1}=p_{2}=0$, we can calculate that the achievable transmission distance declines 11.96%, 26.91%, 43.52%, 60.46% in asymptotic cases and declines 14.39%, 30.93%, 48.56%, 66.19% with the finite-key size when $p_{1}=10^{-6},10^{-5},10^{-4},10^{-3}$. In the Figure 2, compared with the perfect randomness $p_{1}=p_{2}=0$, the achievable transmission distance declines 14.39%, 30.93%, 48.56%, 66.19% in asymptotic cases and declines 15.95%, 31.89%, 48.50%, 65.12% with the finite-key size when $p_{1}=0,10^{-6},10^{-5},10^{-4},10^{-3}$. Nevertheless, we find that the secret key rate can exceed the PLOB bound [11] when $p_{1}\left(p_{2}\right)\geq 10^{-6}$ with the finite-key size $N=10^{15}$, and it can still exceed the PLOB bound [11] when $p_{1}\left(p_{2}\right)\geq 10^{-5}$ in the asymptotic case. From the above calculation data, we can deduce that the fluctuation of the finite-key size is greater than asymptotic cases for the fixed weak randomness parameters. Moreover, comparing with two simulation results, we can find that although the randomness information mastered by Eve of the Figure 2 is twice as much as the randomness information mastered by Eve of the Figure 1, it does not decrease exponentially, which means that once Eve obtains part of the information, it can seriously affect the practical system security.

**Figure 3:**
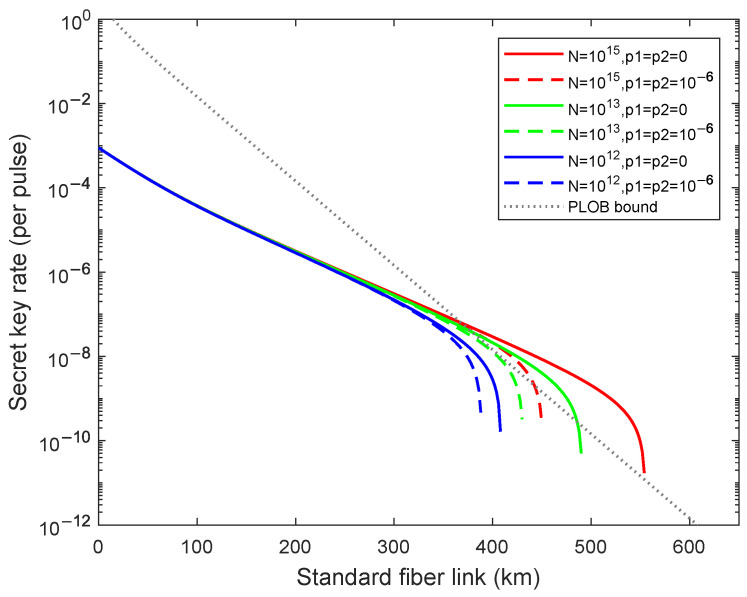
(Color online)The optimal key rate (bits per pulse) in logarithmic scale versus transmission distance between Alice and Bob with weak randomness $p_{1}=p_{2}=10^{-6}$ and without weak randomness $p_{1}=p_{2}=0$ for different $N=10^{x}\left(x=12,13,15\right)$ (curves from left to right), the dashed lines are results of weak randomness for different *N*, and the solid lines are the results of non-weak randomness for different *N*. The gray dotted line is the PLOB bound.

In order to perform a detailed simulation, we then research the results of the weak randomness for the different total numbers of transmitting signals $N=10^{x}\;\left(x=12,13,15\right)$. Corresponding simulation results are illustrated in Figure 3, the dashed lines from left to right are acquired for $N=10^{x}\;\left(x=12,13,15\right)$ with the fixed $p_{1}=p_{2}=10^{-6}$ and the solid lines from left to right are acquired for $N=10^{x}\;\left(x=12,13,15\right)$ with the fixed $p_{1}=p_{2}=0$. We can notice that the generation of the security key rate will be significantly affected, even though the weak randomness parameter is small as $10^{-6}$, which means that Eve will obtain amounts of information even with small proportions of weak randomness. As shown in Figure 3, the achievable transmission distance declines 104 km when $N=10^{15}$, 60 km when $N=10^{13}$, and 10 km when $N=10^{12}$, so we deduce that the greater the number of total pulses, the more the secure transmission distance decreases. We find that the secret key rate cannot exceed the PLOB bound [11] when $N\leq 10^{13}$ with the fixed $p_{1}=p_{2}=10^{-6}$. The number of total pulse increases, so does the number of quantum states which may be attenuated. Eve may obtain more information due to the relation between the expected values and the observed values for the case with different modulated states in the practical QKD system. In this case, the number of modulated states distinguished by Eve may increase which leads to more leakage of the security key information so we are supposed to control the number of total pulses within a rational range rather than arbitrarily choosing.

**Figure 4:**
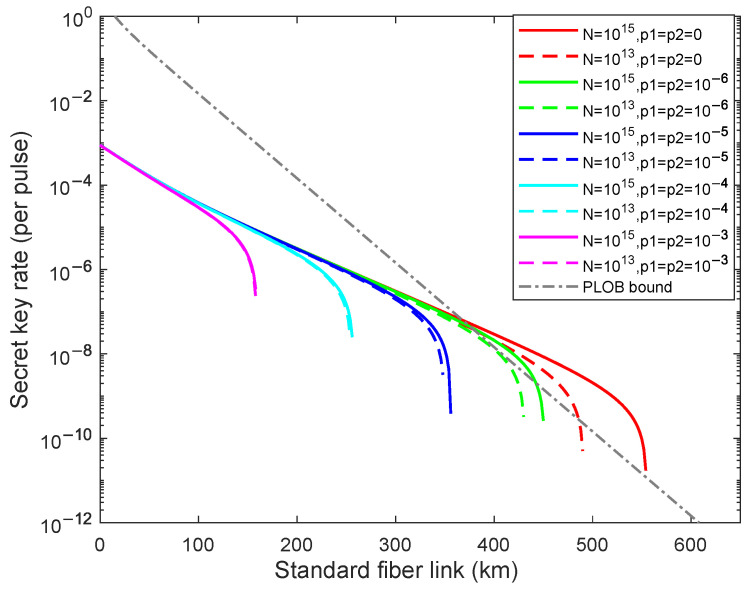
(Color online) The optimal key rate (bits per pulse) in logarithmic scale versus transmission distance between Alice and Bob with weak randomness $p_{1}=p_{2}=0,10^{-x}\;\left(x=6,5,4,3\right)$ (curves from right to left) for different $N=10^{13}$, $N=10^{15}$, the dashed lines are results $N=10^{13}$ with different weak randomness parameters, and the solid lines are the results of $N=10^{15}$ with different weak randomness parameters. The gray dotted line is the PLOB bound.

To further study the impacts of the weak randomness for different total numbers of transmitting signals, we then research the secret key rate for $N=10^{13},\;10^{15}$ with $p_{1}=p_{2}=0,\;10^{-x}\;\left(x=6,5,4,3\right)$ in Figure 4. As illustrated in Figure 4, the dashed lines from right to left are acquired for different weak randomness parameters $p_{1}=p_{2}=0,\;10^{-x}\;\left(x=6,5,4,3\right)$ with the fixed $N=10^{13}$ and the solid lines from right to left are acquired for different weak randomness parameters $p_{1}=p_{2}=0,\;10^{-x}\;\left(x=6,5,4,3\right)$ with the fixed $N=10^{15}$. We can find that the impact of the weak randomness on final security key rate is greater than the finite total numbers of transmitting signals when $p_{1}=p_{2}\geq 10^{-4}$ and the security key rate lines of two different *N* are approximately asymptotic. The impacts of the weak randomness on final security key rate is weaker than the finite total numbers of transmitting signals when $p_{1}=p_{2}\leq 10^{-5}$ and the security key rate lines of two different *N* are not asymptotic. Moreover, the secret key rate cannot exceed the PLOB bound [11] when $p_{1}\left(p_{2}\right)\geq 10^{-6}$ with the finite-key size. In fact, temporal modes of photons become stretched due to the chromatic dispersion in the fiber. This phenomenon will impact the detection time windows. That is, the longer the fiber, the wider the time window should be. The duration of the secret key generation depends on the transmission distance as well as on the number of photons per pulse.

**Figure 5:**
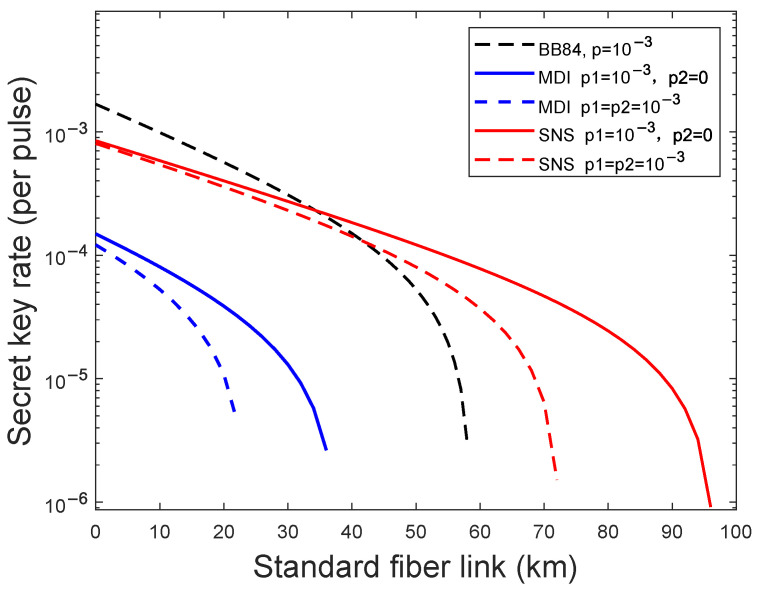
(Color online)The optimal key rate (bits per pulse) in logarithmic scale versus transmission distance between Alice and Bob with weak randomness in the BB84 protocol, the MDI-QKD and the SNS TF-QKD. The blue lines are results weak randomness parameters, and the solid lines are the results of $N=10^{15}$ with different weak randomness parameters.

Finally, we compare and analyze the effects of weak randomness on different QKD protocols, BB84, MDI-QKD and SNS TF-QKD. We simulate the largest weak randomness vulnerability that different protocols can tolerate. As shown in Figure 5, the blue lines are results of MDI-QKD: the solid line is the result of both Alice and Bob existing the weak randomness and the dashed line is the result of one party existing the weak randomness. The black line is the result of the BB84 protocol. The red lines are results of SNS TF-QKD: the solid line is the result of both Alice and Bob existing the weak randomness and the dashed line is the result of one party existing the weak randomness. We find that the largest weak randomness vulnerability that SNS TF-QKD can tolerate is $10^{-2}$ which is greater than the BB84 and MDI-QKD $10^{-3}$. Moreover, the achievable transmission distance is also longer than the BB84 protocol and the MDI-QKD protocol.

Actually, the probability that the states prepared by Alice (Bob) in the BB84 and the MDI-QKD reach the detector is η. For the SNS TF-QKD, it is $\sqrt{\eta }$. Under the weak randomness model, in order to make sure not to be discovered, Eve may attenuate the quantum states from non-random part with a certain probability, which is related to the fiber transmittance η. The channel-loss dependence of the key rate in SNS TF-QKD is square root of channel transmittance $R∼O\sqrt{\eta }$ while it is linear in the BB84 and the MDI-QKD R∼O(η), and that is why SNS TF-QKD can tolerate more weak randomness vulnerability than the BB84 protocol and the MDI-QKD. Compared with the BB84 protocol, the MDI-QKD is more sensitive to weak randomness which is rational since both Alice and Bob prepare quantum states. Compared with the MDI-QKD, the SNS TF-QKD is more robust to the weak randomness since just one party sends states and perform single photon interference in the quantum channel.

From the above simulation results, we can infer that SNS TF-QKD can still have the outstanding performance under the weak random condition. The secret key rate with the finite-key size is more sensitive to the weak randomness, and it performs differently for the different finite numbers of total pulses. Furthermore, SNS TF-QKD has an advantage of tolerance to the weak randomness compared to the BB84 protocol and the MDI-QKD protocol.

## 5. Conclusions

In this paper, we study the influence of weak randomness on the security of SNS TF-QKD in the asymptotic and the finite-key size case. Our simulation results indicate that in both cases, SNS TF-QKD can still have the prominent performance under the weak random condition: the secret key rate can exceed the PLOB bound and achieve long secure transmission distances. Moreover, the fluctuation of the final key rate with the finite-key size is greater than that in asymptotic cases, and because of Eve’s attenuation operation, the greater the number of total pulses, the more reduced the secure transmission distance. Additionally, the impact of the weak randomness on the final security key rate is greater than that with the finite total numbers of transmitting signals when $p_{1}=p_{2}\geq 10^{-4}$, and weaker when $p_{1}=p_{2}\leq 10^{-5}$. Under the weak randomness condition, SNS TF-QKD and MDI-QKD perform differently. The secret key rate of SNS TF-QKD still can surpass the PLOB bound when $p_{1}\left(p_{2}\right)\leq 10^{-6}$ with the finite-key size, and it cannot surpass the PLOB bound when $p_{1}\left(p_{2}\right)\geq 10^{-5}$ in the asymptotic case. MDI-QKD cannot generate a secure key when $p_{1}\left(p_{2}\right)\geq 10^{-3}$, while SNS TF-QKD has an advantage of tolerating the weak randomness (up to $10^{-2}$).

We conclude that to avoid such an attack in the actual QKD systems, two aspects can be taken into account: (1) to make sure that the random numbers we use to encode, select bases, select time windows, and send or not send quantum states are as perfect as possible. We are supposed to use a better random number generator or random number generation algorithm, and (2) at the source side, we should ensure the reduction of the risk of the side channels, so as to avoid the distinguishability of the quantum states preparation in all degrees of freedom, such as the distinguishability between signal states and decoy states, and the distinguishability between perfect random states and weak random states. We can use two independent laser sources so that Alice and Bob have no incidental light, and hence there is no need to monitor the incident light as the implementations directly use seed light from Charlie. The imperfect IM will also produce the states distinguishability in the frequency domain. We can use more than one IM in the actual QKD systems. Furthermore, the narrow spectral filter and wavelength filter can also be used to the reduce states distinguishability and the threat of side channels.

## Figures and Tables

**Table 1:** List of experimental parameters applied in the numerical simulation in the following table: α is the the fiber loss coefficient (dB/km), *f* is the efficiency of error correction, $\eta_{d}$ is the efficiency of the detectors, $e_{d}$ is the probability of the optical misalignment error, $p_{d}$ is the dark count rate, and ε is the failure probability of statistical fluctuation analysis.

α	*f*	$\eta_{d}$	$e_{d}$	$p_{d}$	ε
0.2	1.1	80%	0.15	$1.0\;\times \;10^{-10}$	$1.0\;\times \;10^{-10}$

## Data Availability

Not applicable.
